# Potential Role of Microtubule Stabilizing Agents in Neurodevelopmental Disorders

**DOI:** 10.3390/ijms18081627

**Published:** 2017-07-26

**Authors:** Sara Anna Bonini, Andrea Mastinu, Giulia Ferrari-Toninelli, Maurizio Memo

**Affiliations:** Department of Molecular and Translational Medicine, University of Brescia, 25123 Brescia, Italy; andrea.mastinu@unibs.it (A.M.); giuliaferraritoninelli@yahoo.it (G.F.-T.); maurizio.memo@unibs.it (M.M.)

**Keywords:** neurodevelopmental disorders, microtubule, brain malformation, microtubule stabilizing agents

## Abstract

Neurodevelopmental disorders (NDDs) are characterized by neuroanatomical abnormalities indicative of corticogenesis disturbances. At the basis of NDDs cortical abnormalities, the principal developmental processes involved are cellular proliferation, migration and differentiation. NDDs are also considered “synaptic disorders” since accumulating evidence suggests that NDDs are developmental brain misconnection syndromes characterized by altered connectivity in local circuits and between brain regions. Microtubules and microtubule-associated proteins play a fundamental role in the regulation of basic neurodevelopmental processes, such as neuronal polarization and migration, neuronal branching and synaptogenesis. Here, the role of microtubule dynamics will be elucidated in regulating several neurodevelopmental steps. Furthermore, the correlation between abnormalities in microtubule dynamics and some NDDs will be described. Finally, we will discuss the potential use of microtubule stabilizing agents as a new pharmacological intervention for NDDs treatment.

## 1. Introduction

Neurodevelopmental disorders (NDDs) are a group of heterogeneous conditions linked to impaired brain development leading to emotional, relational and cognitive deficits. They include different pathologies, such as autism spectrum disorders, schizophrenia and epilepsy. Owing to the lack of knowledge of the pathogenic mechanisms, no current therapy exists for rescue or prevention of NDDs. Drugs currently used for NDDs are mainly symptomatic (antipsychotic medications combined with psychosocial treatment), lack specificity and are often ineffective. NDDs are characterized by neuroanatomical abnormalities that can include variation in total minicolumnar number in the cortex [1,2,3], altered cortical folding [4], various cortical and subcortical heterotopias and dysplasias; these abnormalities are indicative of corticogenesis disturbances, finally causing disruption of cortical laminar architecture [5,6]. Several developmental processes are involved in NDDs-linked altered cortical development, such as cellular proliferation, migration and differentiation. Accumulating evidence sustains the concept of NDDs as “synaptic disorder” due to the alteration in both local and global brain connectivity.

Data from post-mortem brains suggest altered neuron production and migration, disrupted synaptogenesis of both excitatory and inhibitory synapses, leading to faulty assembly of microcircuitry in multiple brain regions [7]. Microtubules (MTs) hold a fundamental role in regulating all the steps of neurons and therefore brain development. In the present review we will discuss the importance of MTs in neuron lifecycle regulation, how MTs genetic or functional alterations are linked to different NDDs and the potential use of MTs stabilizing agents as new therapeutic approach in NDDs.

### 1.1. MTs Role in Neuron Lifecycle

**Figure 1:**
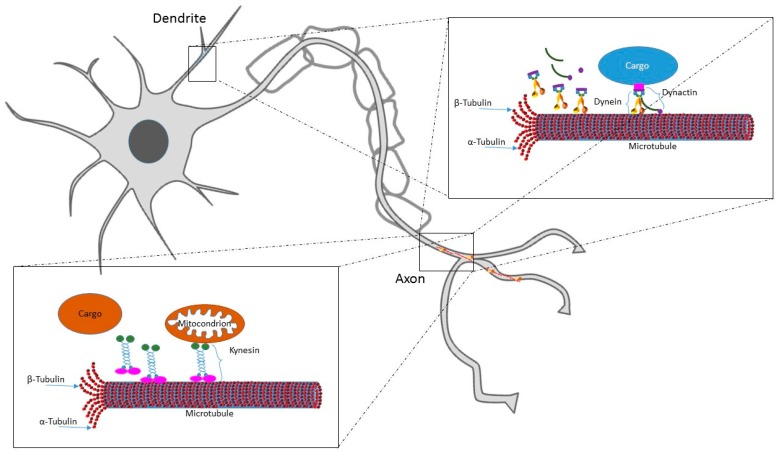
Scheme of MTs dynamics and trafficking.

MTs are basic elements of the cytoskeleton composed of α- and β-tubulin heterodimers. Together with actin microfilaments and intermediate filaments (called neurofilaments in neurons), they constitute the cytoskeleton, the dynamic structure that gives the cell its shape and mechanical resistance to deformation. α- and β-tubulin form linear protofilaments, the basis of MTs polar structure, where β-tubulin monomer is pointing towards the fast growing “plus-end”, and α-tubulin is located at the slower growing “minus-end” [8]. The MTs plus-end grows out from the centrosome and undergoes rapid switches from growth to shrinkage (called “catastrophe”) and from shrinkage to growth (called “rescue”) [9]. On the other hand, MTs minus-end is firmly attached to the centrosome. MTs are distributed in the whole cell body and form a dynamic network that incessantly growth, retracts, warps and breaks. Hence rather than providing cell rigidity, they have an important role in running dynamic processes, including intracellular cargo transport along the axons and mitotic spindle formation; these cellular events are completely dependent on MTs ability to be polymerized, depolymerized and severed [10,11]. Indeed, they provide platforms for intracellular transport of secretory vesicles and organelles. A schematic representation of MTs dynamics and trafficking is reported in Figure 1. MTs are nucleated and organized by the centrosome, the main cytoskeleton-organizing center in the cell. The centrosome has a crucial role for coordinating several steps of neuron formation and maturation, such as proliferation, migration, and differentiation [12]. The centrosome is the main MTs nucleation site in most animal cell, however MTs nucleation can occur also via centrosome-independent processes [13]. In the phase of neuronal polarization, in particular during axon specification, MTs are nucleated at the centrosome and regularly localized in all the cell neurites. Successively, at later stages of neuronal development, MTs maintain their uniform orientation in the axon whereas they lose it in the dendrites, where they acquire a mixed orientation. In this phase, acentrosomal MTs assembly takes place. In particular, γ-tubulin has been demonstrated to play the role of MTs local nucleator in developing neurons; it is present both in axon and dendrites and organizes the cytoskeleton during neuronal differentiation and plasticity [14].

In first steps of neurodevelopment, cortical neurons are produced by neurogenesis in progenitor zones adjacent to lateral ventricles. During cell proliferation, also the centrosome splits in two newly formed centrosomes that coordinate the assembly and organization of the mitotic spindle [15]. Just after division, neurons are often characterized by a multipolar morphology that only subsequently switches to a bipolar morphology. This transition is caused by the asymmetric transfer of the centrosome to the site in which a membrane protrusion (the leading process) is extended [16]. Following cell proliferation, postmitotic neurons start to migrate to form the complex but highly specified cortical structure, organized in six horizontal layers and vertically oriented minicolumns [17]. The two main classes of cortical neurons are produced in different brain regions and migrate differently: projection neurons originate in the cortical neuroepithelium (pallium) and migrate mostly along the radial axis to cortical plate, whereas interneurons are produced in the basal telencephalon (ganglionic eminences) and subsequently migrate mostly tangentially to their final cortical destination [18]. The overall process of neuronal migration needs a high degree of cell motility and morphological flexibility; it consists of four interrelated but independent biological events: polarization, protrusion, adhesion and retraction [19]. The migration process is coordinated by both intracellular and extracellular signaling mechanisms, the latter coming from the extracellular matrix (ECM). Centrosome, MTs and actin play a critical role in regulating this cell event. As a first step the neuron needs to polarize in a certain direction, forming a leading process, a cytoplasmic swelling and a trailing process. Then, both the nucleus and the soma move following the leading process, that extends and retracts along radial glial cells, the tracking guide of radially migrating neurons, by means of motor proteins. The centrosome is localized between the nucleus and the leading process; nucleus and centrosome are linked together by perinuclear MTs in a cage-like structure [20] that elongate also in the leading process. Centrosome moves towards the leading process and carry the nucleus along the MTs network (nucleokinesis). In particular, MT tip binding proteins adenomatous polyposis coli (APC) and MT actin crosslinking factor 1 (MACF1) are crucial for centrosome forward movement and neuronal migration in the developing neocortex. APC is a MT anchoring protein that, following glycogen synthase kinase 3 (GSK3) inactivation by liver kinase B1 (LKB1), localizes at the distal ends of MTs in the leading process tip, and this consequently leads to MT stabilization [21]. MACF1 also interacts with GSK3 and mediates MT stability [22]. Several proteins, called cytoskeletal motor proteins, regulate neuronal migration and cellular cargos axonal transport by binding and moving along MTs. They include the dynein family, proteins that move towards the minus-end of MTs (retrograde transport), and kinesin family, proteins that move towards the MTs plus-end (anterograde transport). Lis1, together with NudE and NudEL, is a dynein regulatory factor that stimulates dynein motor activity and pulls captured MTs and the centrosome into the swellings that appear transiently within the leading process [12].

The ECM signals define the timing and the final destination positioning of migrating neurons. Cytoskeleton and ECM work together to coordinate all the migration process steps. Failure to reach the correct final positioning cause neuron ectopic localization leading to abnormal laminar cytoarchitecture and cortical disorganization. This migration failure is at the basis of anatomical and functional defects of several NDDs, including lissencephaly, schizophrenia, autism and intellectual disability [23].

During migration, most immature neurons polarize and develop an axon and several dendrites from the leading and the trailing processes [24]. Differentiated neurons establish synaptic contacts by means of axon and dendrites in order to create a complex and highly efficient functional connectivity network. In the axon usually MTs are long and homogeneously oriented, with the plus-end towards facing outward. On the contrary, in the dendrites MTs are oriented in both directions, have different post-translational modifications and cytoskeletal density, other than MTs associated proteins [25]. Specific post-translational modifications, including acetylation, detyrosination and polyglutamilation, occur after MTs polymerization and accumulate in a subgroup of more stable MTs in an age-dependent manner [26]. Instead, tyrosination is a marker of more instable MTs [27,28]. The opposite action of stabilizing proteins and MTs severing proteins maintain the dynamics of MTs [29,30]. Tau, a protein of the MTs associated proteins (MAPs) family, belongs to the first group, whereas katanin and spastin, proteins that promote MTs remodeling required for neurites outgrowth and branching, to the second [25,31].

MTs severing proteins cause local MTs disruption in axons and dendrites and play a role in MTs rearrangements, necessary for neuronal differentiation and plasticity.

Synaptic activity also has emerged as one of the factors able to regulate MTs modifications [32]. In particular, dopamine has been shown to influence interneurons tangential migration by redistributing cytoskeletal elements [33], and gamma-aminobutyric acid (GABA) has been demonstrated to act as a chemoattractant for migrating neurons [34]. In this way, MTs modifications are closely related to extracellular signals and intracellular cargo trafficking.

Proper regulation of MTs dynamics is essential for neuron functionality. Indeed, to function properly neurons require a well stabilized MTs network and an efficient axonal transport system. If these conditions are not satisfied, neurons degenerate [35]. Altered regulation of MTs dynamics could have deleterious effects on cell viability; MTs can be considered as “biosensors” of cellular wellbeing [36].

### 1.2. Pathways Involved in MTs Dynamics Regulation

MTs dynamics is spatially and temporally regulated by several pathways and MTs-interacting proteins [11,23]. Here we will describe some of the pathways known to have a role in orchestrating MTs dynamic remodeling.

Rho-associated coiled-coil kinase (ROCK) pathway is composed by two kinases: Rho-associated coiled-coil kinase 1 and 2. ROCK phosphorylates several substrates, including the actin regulatory proteins myosin light chain [37], the LIM kinases [38], and the intermediate filament proteins desmin [39] and vimentin [40]; it plays essential roles in promoting actomyosin cytoskeleton contractility downstream of RhoA and RhoC activation [41]. Furthermore, it regulates MTs acetilation by phosphorylating the tubulin polymerization promoting protein 1 (TPPP1/p25) [42]. TPPP1 phosphorylation by ROCK promote cell motility and migration. ROCK pathway plays vital roles during development regulating several embryonic morphogenetic events, such as cell migration, differentiation, and axis formation [43].

(aPKC)-Aurora A-NDEL1 pathway regulates MTs dynamics during neurite elongation [44]. The phosphorylation of Aurora A, an integral mitotic kinase, by an atypical protein kinase C (aPKC), followed by phosphorylation and recruitment of NDEL1, is essential for neurite extension in most-mitotic neurons [45].

Wnt-dishevelled pathway increases MTs stability in the axon and induces the formation of looped MTs at enlarged growth cones by concomitant inhibiting Gsk3β and activating c-Jun N-terminal kinase [46]. Gsk3, a major downstream component of the Wnt, is a critical kinase for MTs regulation during neural development. Indeed it is present along the length of spindle MTs and regulates both centrosome orientation and MTs spindle formation during cell division [47,48]. Gsk3 phosphorylation on serine 9 by protein kinase B (PKB) causes its inactivation and consequent suppression of mitotic chromosome movement; this leads to a prometaphase-like arrest [49]. Wnt signaling regulates many developmental processes, including cell proliferation, specification, differentiation and migration. It also regulates neuronal differentiation of cortical intermediate progenitors [49]. Li and colleagues demonstrated that Wnt5, a morphogen of the Wnt protein family, is able to increase axon outgrowth by creating and reorganizing dynamic MTs that form bundled array oriented in the direction of axon extension [50]. Wnt5 gradients induce asymmetric redistribution of dynamic MTs toward the far side of the growth cone and evoke calcium transfer. The Wnt/calcium signaling is mediated by tau, the MTs associated and stabilizing protein, and has emerged to have an important role for MTs reorganization during growth cone remodeling. Wnt, together with the Notch pathway, determines the cellular fate during brain development; the alteration of these two pathways during neurodevelopment might participate to the onset of focal cortical dysplasia (FCD). FCD is a disorder characterized by cortical laminar disorganization, heterotopic white matter neurons, abnormal large neurons and balloon cells, due to abnormalities of neuronal migration and differentiation [51].

Reelin pathway has a key role in assembly of cortical cytoarchitecture. Reelin is an extracellular molecule secreted by Cajal-Retzius cells that when binding to its receptors causes a series of phosphorylated signaling cascade leading to MTs dynamics regulation. Reelin has a main role in proper positioning of migrating neurons, indeed it promotes neuronal detachment from radial glial cells [52]. Disruption in the Reelin signaling pathway causes disorganized cortical lamination in mice and severe NDDs in human, such as lissencephaly and cerebellar hypoplasia [53], schizophrenia, bipolar disorder and autism [54].

Notch pathway, a key regulator of neural stem cells and neural development, affects neuronal migration by altering the morphology of migrating neurons. Notch signaling activation leads to a bipolar morphology that favors migration. A cross-talk between Notch and Reelin pathways does exist and is required for coordinating cortical neurons migration [55,56]. Notch pathway is a key regulator of neuronal structural plasticity by remodeling cytoskeleton. Indeed, activation of Notch pathway in primary cortical neurons results in reduced neurite branches and loss of varicosities, and in increased α-tubulin acetylation and poly-glutamilation that reflect MTs stability [57]. Notch pathway MTs remodeling effect is also mediated by spastin, indeed activation of Notch pathway reduces spastin mRNA and protein levels, further increasing MTs stability [58]. These are dynamic events that can be reversed by inhibition of Notch pathway.

NF-κB pathway, in addition to its role in regulation of immune and inflammatory response, acts on neurons remodeling. NF-κB is composed by five intracellular subunits (p50, p52, c-Rel, p65 and Rel-B) that, once activated, can form homo- or heterodimers and promote gene transcription [59]. Recently it is emerged a role for NF-κB pathway in neuronal structural plasticity regulation [60]. During development NF-κB signaling has a role in the control of axon initiation, elongation, guidance and branching, and in the regulation of dendrite arbor size and complexity. In adult neurons it regulates dendritic spine density. It has been demonstrated that dephosphorylation of NF-κB p65 subunit is required for the BDNF-promoted neurite outgrowth [61]. Furthermore, lack of p50 subunit results in reduced neurite branching and varicosities loss, and these events are Notch pathway-mediated [62]. In mice p50 knock-out cortical structure alterations have been found, with an increase in specific layers’ thickness and impaired columnar organization [63]. The mechanisms through which NF-κB exerts its role in cytoskeleton remodeling are still largely unknown, but it has been demonstrated that MTs associated proteins MAP2 and MAP1B, essential proteins for neurites elongation, are NF-κB transcriptional targets [64]. Moreover, a direct cross-talk between NF-κB and Notch pathways in cortical neurons has been found [62].

## 2. MTs-Linked NDDs

The complex and plastic morphology of neurons, both during migration and during establishment of their dendritic and axonal arbors to form synaptic contacts, implies a strictly regulated process of cytoskeletal structuring. Therefore, it is consistent the causal link between deficiencies in cytoskeletal protein-encoding genes and several NDDs. Mutations in genes encoding centrosomal proteins cause severe NDDs that lead to several neuropsychiatric disorders, such as lissencephaly, microcephaly and schizophrenia [12]. Also mutations in the various α- and β-tubulin genes, which encode for the globular proteins that polymerize into MTs structures, have been associated to NDDs [65]. Here below we will describe some of the neurological disorders that have been linked to alterations in MTs regulatory genes.

### 2.1. Autism

Autism, more properly defined as autism spectrum disorders (ASD), comprises a heterogeneous group of neurodevelopmental disorders characterized by deficits in social interaction, verbal and non-verbal communication, restrictive interests and repetitive behaviors. ASD are characterized by neuroanatomical abnormalities with alterations in minicolumnar and laminar cortical organization, synaptic disorders and misconnection in neuronal brain circuitries. At the basis of several ASD-linked structural brain abnormalities, different MTs-associated gene mutations and alterations exist. A summary of MTs regulatory protein and gene alterations associated with ASD has been reported in Table 1.

One of these is the *ADNP* mutation [66]; activity-dependent neuroprotective protein (ADNP) is fundamental for brain formation and function, indeed it is involved in cell differentiation and maturation. It is a member of the chromatin remodeling complex SWI/SNF also associated with tau alternative splicing [66]. Illana Gozes and colleagues recently found an ADNP cytoplasmic interaction with the MTs end-binding (EB) proteins [67]. The ADNP-belonging NAP neuroprotective peptide binds directly MTs and is implicated in synaptic plasticity regulation, acting as neurotrophic factor. Indeed, NAP is able both to bind EB1 to enhance neurite outgrowth and to bind EB3 to enhance post-synaptic density 95 protein (PSD-95), thus modulating dendritic plasticity. It has also been shown to recruit tau to MTs [68] hence promoting axonal transport [69].

Slit/Robo cell signaling pathway has been found altered in ASD patients [70]. It is composed by three glycoproteins (Slit 1–3) that interact with the repulsive guidance transmembrane receptors Robo (Robo 1–4). Slit/Robo pathway regulates cytoskeletal remodeling acting on dendritic branching and axon pathfinding. In ASD patients increased expression of *Slit 1* and decreased expression of the *ABL1* and *Cdc42* genes have been found. ABL1 is a kinase required for the activation of Cdc42, a component of the Rho family of GTPases essential for neurite outgrowth [71].

Few years ago, Berg and colleagues found altered expression of Janus kinase and microtubule-interacting protein 1 (JAKMIP1) in patients with distinct syndromic forms of ASD [72]. JAKMIP1 is expressed in both neuronal and lymphoid tissues and comprises two distinct functional domains: a C-terminal regulatory region, and an N-terminal region that targets the protein to MTs polymers; its overexpression in human cell lines profoundly perturbs MTs network inducing the formation of tight and stable boundles [73]. It probably is involved in several cell dynamic processes, such as polarization, segregation of signaling complexes and vesicle traffic. JAKMIP1 is a component of polyribosomes and a ribonucleoprotein translational regulatory complex. JAKMIP1 loss dysregulates neuronal translation during synaptogenesis altering glutamatergic NMDAR signaling and causes social behavior impairments, stereotyped activity, abnormal pups vocalizations in mice.

The MTs-associated stable tubule only polypeptide (STOP) protein, also known as MTs-associated protein 6 (MAP6), is a MTs stabilizing protein that plays regulatory roles in neurons, such as regulation of both actin and MTs dynamic in axons, dendrites as well as in dendritic spines and synaptic protein complexes [74]. STOP/MAP6 protein has emerged to be significantly reduced in the cortices of BTBR mouse brain, a validated animal model of ASD [75]. The authors also demonstrated significant reduced protein levels in the plasma of autistic subject compared to healthy controls [76]. STOP/MAP6 knock-out mice display several synaptic abnormalities and behavioral deficits, including impairments in maternal care and social interaction [77], and alterations in mood and cognitive performances [78].

Liu and colleagues [79] demonstrated that KIRREL3, a synaptic molecule of the immunoglobuline superfamily, implicated in several NDDs including ASD [80], interacts with the brain expressed protein MAP1B, the MTs-associated protein 1B. MAP1B plays important roles in the regulation of neuronal morphogenesis. Its deficiency causes abnormal actin microfilament polymerization and altered activity of GTPases that regulate actin cytoskeleton [81]. MAP1B^−/−^ mice showed impaired long term potentiation and altered locomotor activity [82].

Autism susceptibility candidate 2 (*AUTS2*), a gene linked to several psychiatric diseases including ASD [83], regulates neuronal migration interacting with several MTs related proteins [84]. Indeed, the protein AUTS2 interacts with guanine nucleotide exchange factors (GEFs), P-Rex-1 and Elmo2/Dock180 complex to activate Rac1, a Rho family small GTPase, that coordinates actin polymerization and MTs dynamics [85]. Furthermore, AUTS2 interacts with other GEFs, intersectin 1 and 2, to suppress the activities of Cdc42, another Rho family GTPase, thus repressing filopodia formation in neuronal neuritis and cell body. *AUTS2* mut neurons display abnormal morphology during migration and reduced activity of JNK, a protein that under the control of Rac1 is involved in leading the process formation of migrating neurons through the regulation of MTs dynamics [86]. So alteration of the AUTS2/Rac1 pathway cause impairment in neuronal migration by deregulating actin and MTs dynamics in the cytoskeleton [84].

Duplication of the 14-3-3epsilon (*YWHAE*) gene has been recently associated to ASD onset in human patients [87]. Cornell and colleagues [88] demonstrated that YWHAE binds to the MTs binding protein doublecortin (Dcx) preventing its degradation. Increased levels of Dcx disrupt neurite formation by preventing the invasion of MTs into lamellipodia. On the contrary, mice lacking 14-3-3epsilon result in increased neurite formation. 

The partitioning-defective 1 (Par1) family of kinases, or MTs affinity-regulating kinases (MARKs), has an important role in establishing cell polarity, regulate vesicular transport and cell migration [89]. Dysregulation of these kinases has been linked to ASD [90]. In particular, several single-nucleotide polymorphisms (SNPs) in *MARK1* gene have been found associated to autism [91]. MARKs phosphorylate tau protein and related MTs-associated proteins (MAPs) thus regulating MTs dynamics in neurons.

Recent studies on de novo mutation associated to ASD by sequencing the human exomes identified *KATNAL2* gene mutation as a risk factor for the disease [92]. Katnal2 is a MTs severing ATPase and *KATNAL2* gene deletion in mouse resulted in both reduced number of branches and length in developing neurons [93].

### 2.2. Schizophrenia

Schizophrenia is a psychiatric disorder with high disability and distress that can occur in adolescence or early adulthood [94]. Schizophrenia pathological features include auditory and visual hallucinations, decreased sensory gating, reduced social interactions, and working memory deficits [95]. All antipsychotic drugs counteract positive symptoms by antagonizing dopaminergic D2 receptor, nonetheless these compounds show poor efficacy for cognitive and negative symptoms [94,96]. Currently, schizophrenia can be considered a NDD, due to defects during neurodevelopment leading to abnormal brain cytoarchitecture [97]. Indeed, schizophrenia is associated with brain structural changes such as synaptic pruning defects, spine and dendrite atrophy [98]. Also, susceptibility and onset of schizophrenia is strongly related to brain plasticity alteration characterized by reduced pruning and spinogenesis defects in the cortex [99]. Cytoskeleton dysfunctions has been inferred in the pathology of schizophrenia. In particular, tubulin isotypes expression is significantly altered in clinical and in experimental models of schizophrenia, indicating that perturbed MTs homeostasis is a hallmark in this pathology [100].

Tubulin displays heterogeneous structures and differential post-translational modifications within the C-terminal domain, temporally regulated during brain development [101]. Several authors have associated some tubulin post-translational alterations to schizophrenia [102]. Tubulin acetylations and deacetylations are post-translational modifications that occur within the MTs lumen catalyzed by acetyl transferases (αTAT) and histone deacetylase 6 (HDAC6) respectively [102]. Interestingly, *Ulk4* gene (Unc-51 like kinase-4) is disrupted in schizophrenic patients. Knockdown of *Ulk4* gene showed a diminished levels of acetylated α-tubulin suggesting destabilized MTs [103]. Moreover, knockdown of Ulk4 leads to decrease of c-Jun N-terminal kinases (JNK) activity, a major regulator of MTs homeostasis, and reduced dendrite length, branching, and agenesis of the corpus callosum [103].

Another post-translational modification is α- and β-tubulin glutamylation by enzymes belonging to the tubulin tyrosine ligase-like (TTLL) family [104,105]. Molecular interactions between tubulin, MAPs and motor proteins are influenced by polyglutamylation-dependent changes in the surface charge [104]. Several authors proposed that alterations in tubulin polyglutamylation could contribute to the onset and development of schizophrenia [106]. Healthy neuronal function and network formation are crucial events that are based on the normal cytoskeletal organization and dendritic arborization regulated by MAPs. In fact, MAP2 immunoreactivity is reduced in the hippocampus and prefrontal and auditory cortex of post mortem tissues of schizophrenia patients [107,108]. Another MTs regulatory protein involved in schizophrenia is MAP6. MAP6 stabilizes adjacent MTs through the formation of bridges between them and this mechanism is regulated by calmodulin binding [109]. Moreover, several features of murine models associated with schizophrenia such as anxiety, cognitive deficits, hyperactivity, social impairments and glutamatergic transmission are originated by genetic deletion of *MAP6* [78].

*DISC-1* is another gene involved in cytoskeletal remodeling and its alteration is associated to schizophrenia [110]. Interaction of the nuclear distribution element-like protein (NDEL1) and pericentriolar material-1 protein with *DISC-1* regulates axonal transport and MTs organization [110]. Moreover, the dynein motor complex anchoring at the centrosome is regulated by interaction between *DISC-1* with NDE1 and NDEL1 and this molecular mechanism may be connected to LIS1. Genetic association studies have identified a single nucleotide polymorphism (SNP) in *DISC-1* gene, which increases the risk of developing schizophrenia. Interestingly, this DISC-1-SNP variant fails to interact with NDEL1, indicating that the MTs cytoskeleton disruption contributes to the pathology development [111]. 

The activity-dependent neuroprotective protein (ADNP) has been found deregulated in postmortem hippocampi of schizophrenia patients [112]. ADNP directly interacts with the MTs associated protein 1 light chain 3 (LC3) that regulates autophagy [113].

Lastly, collapsin response mediator protein-2 (CRMP-2) is a tubulin binding protein that is enriched in the hippocampus and dentate gyrus of adult brain [114]. *CRMP-2* gene is within a schizophrenia susceptibility region on chromosome 8p21 [115]. Post-mortem brain analysis from individuals with schizophrenia showed an increased expression of CRMP2 by genetic linkage data and proteome-wide analysis [116]. Several MTs and MTs regulatory protein and gene alterations associated with schizophrenia are listed in Table 2 (table modified from [102]).

### 2.3. Down Syndrome

Trisomy 21 (Down syndrome, DS) is a pathological condition associated with mental retardation and represents the most common genetic cause of developmental disability [117]. Fetal DS brain show a delay in cortical development and a disorganized lamination [118]. Moreover, DS brain present dendrite alterations with decreased number of spines and altered morphology and defective cortical layering [119]. Cortical cytoarchitecture alterations in DS are characterized by cytoskeletal abnormalities in prenatal life. In particular, β-tubulin expression is reduced in DS and this condition, correlated with the changes in synaptogenesis, can affect MTs-based functions, ranging from neuronal migration to intracellular transport [120]. Also components of the dynactin complex are significantly reduced in fetal DS brain [121]. Disruption of dynein/dynactin proteins structure and functionality cause synaptic instability and onset of neuronal degeneration associated with DS [121,122].

Interestingly, another cytoskeletal abnormality associated to neuropathological features of DS is linked to dual-specificity tyrosine-phosphorylation-regulated kinase 1A (*DYRK1A*) [123]. This gene is located on critical region of the chromosome 21 containing a subset of genes implicated in trisomy development [124]. Moreover, DYRK1A expression in the brain tissue of DS subjects is increased and its up-regulation has been linked to brain anomalies and deficiencies in complex neuronal networks [125]. The pathogenesis of mental retardation in DS is associated to the formation of defective neuronal circuits and generated by an increased expression of DYRK1A [124]. Indeed, DYRK1A overexpression causes a reduction in dendritic spine formation of cultured hippocampal neurons [126]. Moreover, cortical neurons from DYRK1A transgenic mice displayed a reduction in spine density, synapse formation, and dendritic filopodia length as well as alteration in spine morphology [127]. These anatomical anomalies DYRK1A-mediated in the brain of DS subjects involves the dysregulation of cytoskeletal proteins such as tubulin, actin, and MTs-associated protein [128]. Indeed, DYRK1A regulates MTs growth through phosphorylation of β-tubulin, and this action has a profound influence not only on dendritic arborization, but also on neuron functionality [127]. DYRK1A overexpression together with post-translational MTs alterations, could provide insight into the nature of the neuronal pathologies in DS.

### 2.4. Epilepsy

Epilepsy is a common and chronic neurological disorder characterized by recurring, uncontrolled seizures and often requires long term anti-epileptic drugs therapy [129]. Approximately 30% of epilepsy cases are refractory to current pharmacological treatments. Malformations of cortical structure linked to genetic conditions are at the basis of several forms of epilepsy [130].

One of the gene found to be linked to epilepsy is *DCX*, the gene coding for the doublecortin protein. Kim and colleagues reported a novel missense mutation of *DCX* resulting in late-childhood-onset familiar focal epilepsy and anterior dominant pachygyria without subcortical band heterotopia in both genders [131]. DCX is a MTs-associated protein expressed by neuronal precursor cells and immature neurons in embryonic and adult cortex. It has an important role for MTs stabilization during neuronal migration [132].

Increased expression levels of EMAP-like protein 5 (EML5), a new echinoderm MTs-associated protein expressed in the central nervous system, have been found in both neurons and glial cells of the anterior temporal cortex of intractable epilepsy patients [133]. Repetitive brain seizures induce overexpression of cytoarchitectural-related proteins at the seizure lesion and contribute to cell death, which finally lead to drug resistance. EMAP proteins play a role in MTs formation, depolymerization, extension inhibition and MTs dynamics regulation [134]. They act as MTs disassembly proteins. Overexpression of EML5 found in the anterior temporal cortex of intractable epilepsy patients could be responsible for the increased extension of neuronal dendrites and axons and enhanced branching.

Mesial temporal lobe epilepsy (MTLE) is another common form of intractable epilepsy characterized by cytoarchitectural abnormalities, neuronal loss and reactive gliosis [135]. Several cytoskeletal proteins, such as β-tubulin, tubulin α-1 chain and neuronal tropomodulin profiling II, have been found markedly reduced in MTLE. On the contrary, the growth cone associated protein GAP43, MAP2 and MAP1A have been found significantly increased [136,137,138]. Also the laminin-β1 and integrin α-2 proteins expression levels have been reported to be differentially regulated in chronic epilepsy [139]. Laminin-β1 is an extracellular matrix component that promotes neuronal polarization via regulating directional MTs assembly through β1-integrin [140]. Integrins are transmembrane receptors connecting the extracellular matrix environment to the cytoskeleton; they control cell motility, proliferation, survival and polarization [141]. Finally, the role of MTs dynamics in epilepsy has been analyzed and it emerged that expression of tyrosinated tubulin, a reflection of MTs dynamic properties, is increased in epileptic foci [142].

These data reinforce the concept of abnormalities in cytoskeletal proteins leading to structural alterations in the brain of patients with epilepsy.

### 2.5. Lissencephaly

Lissencephaly, also called agyria/pachygyria, is a neurological disorder in which the mature brain is deficient in gyration [130]. Indeed, it is characterized by reduced or absent gyration combined with thickening of the cerebral cortex. Based on brain histology, lissenchepaly can be classified as type I, or classical lissencephaly, in which the cortex presents only four layers, and type II, or cobblestone lissencephaly, in which no discrete cortical layers are formed. Patients suffering from lissenchepaly (smooth brain) harbor cortical malformations caused by impaired radial neuronal migration and failure of cortical fold formation. These cortical structural alterations cause severe deficits, such as mental retardation, seizures and also death in early childhood. Approximately in the 80% of the lissencephaly cases a genetic cause can be found. Several genes associated to lissencephaly code for proteins that play important roles in brain development, acting on formation and regulation of MTs. Here we will describe the main genes involved in the pathology. A summary of MTs regulatory protein and gene alterations associated with lissencephaly has been reported in Table 3.

The loss of lissencephaly-1 (LIS1) is a major cause of lissencephaly, leading to abnormal nuclear translocation during neuronal migration and hence impairing brain gyrus formation. Cortical malformations are localized more in the dorsal portion of the brain. LIS1 protein is involved in MTs regulation and MTs-based motor proteins [143]. Following phosphorylation, it interacts with tubulin and modulates MTs dynamics, hence is important for preserving MTs network organization [144]. Furthermore, LIS1 interacts with several MTs-associated proteins (MAPs), such as DCX, CLIP-170 and MAP1b, other than with cytoskeletal motor proteins, such as dynein/dynactin, suggesting also a role in regulating intracellular transport, organization of intracellular organelles and mitosis and MTs polarity. LIS1 was found to be essential for radial glial progenitor cells mitotic spindle orientation [145].

Another common gene mutation associated to lissenchepaly is linked to the *DCX* gene. *DCX* gene mutations cause cortical structural alterations mainly localized in the rostral part of the brain [146]. Often in female with *DCX* gene mutations the subcortical band heterotopia (SBH) occurs [147]. SBH is a lissencephaly related disorder characterized by bilateral bands of gray matter interposed in the white matter between the cortex and the lateral ventricles. DCX is a MTs-associated protein that promotes nucleation, assembly and stability of 13-protofilament MTs [148]. DCX-MTs interaction is mainly regulated by DCX phosphorylation by different kinases that can have different effects on interaction affinity. DCX also regulates actin cytoskeleton thus affecting also neurite branching [149,150].

Mutations in the *TUBA1A* gene, coding for the tubulin α1A protein, have been associated to type I lissencephaly [151,152]. TUBA1A protein is a major component of MTs. Patients with TUBA1A mutations present not only cortical dysgenesis, but also cerebellar, hippocampal, corpus callosum, and brainstem abnormalities.

Mutations in *TUBB2B* gene, coding for tubulin β-2B chain, cause asymmetrical bilateral polygyria [153]. Polymicrogyria present disorganized cortical lamination and the presence of multiple small, partially fused gyri separated by shallow sulci that result in an irregular cortical surface.

An autosomal recessive form of lissencephaly has been associated with the human *RELN* gene mutations. It is characterized by severe abnormalities of the cerebellum, hippocampus and brainstem. The mutations disrupt RELN cDNA splicing, resulting in low or undetectable amounts of reelin protein [53]. Reelin is a large secreted glycoprotein synthesized by Cajal-Retzius cells in the marginal zone of the cortex that plays a fundamental role in neuronal migration and positioning in the developing brain by controlling cell-cell interactions. It also regulates MTs dynamics during neuronal migration. Indeed, reelin induces stabilization of the leading processes by binding to migrating neuron ApoER2 receptors. This event involves adaptor protein disabled 1 (Dab1) phosphorylation, adhesion molecule expression, cofilin hosphorylation and inhibition of tau phosphorylation [154].

### 2.6. Microcephaly

Microcephaly is a neurological disorder characterized by reduced prenatal brain growth without structure alterations. Affected patients generally present striking neurological defects and seizures, other than severely impaired intellectual development and disturbances in motor functions. Altered neurogenesis during neurodevelopment is the main cause of microcephaly. Most of microcephaly cases are linked to genetic alterations.

So far, five genes associated with autosomal recessive primary microcephaly (MCPH) have been identified in patients: *MCPH1*, *CDK5RAP2*, *ASPM*, *CENPJ*, and *STIL*/*SIL* [155,156]. All these genes code for proteins localized in the centrosome that play important roles in centrosome formation and stabilization, or regulation of MTs dynamics. Disruption of one of these functions cause dramatic effects in prenatal neurogenesis finally leading to microcephaly.

Recent genetic studies identified biallelic mutations of *PRUNE1* gene in microcephaly affected patients [157]. PRUNE is a member of the DHH (Asp-His-His) phosphoesterase protein superfamily of molecules important for cell motility. Mutations in *PRUNE1* gene caused impaired MTs polymerization, as well as cell migration and proliferation defects. PRUNE may have a role in MTs polymerization regulation, essential process for the cytoskeletal remodeling that occur during cellular division and proliferation.

A partial duplication of *LIS1* has been also associated to microcephaly characterized by neurodevelopmental delays, profound white matter atrophy and in the absence of overt lissencephaly [158].

McNeely and colleagues demonstrated in a mouse model lacking the Kif20b protein, that Kif20b is required for proper morphogenesis of neurons during corticogenesis [159]. Loss of Kif20b causes altered neurite polarization, outgrowth, branching and caliber, other than thalamocortical axon guidance defect and microcephaly. Kif20b, also called MPP1 or Mphosph1, is a member of the Kinesin-6 family of “mitotic” kinesins. Authors suggest that Kif20b protein act by bundling MTs into tight arrays and by localizing effectors.

### 2.7. Intellectual Disability

Intellectual disability (ID), or mental retardation, is a condition included in NDDs that affects 1.5–2% of general population [160]. ID can be classified into syndromic, when intellectual deficits are associated to other medical and behavioural symptoms, or non-syndromic, when only intellectual deficits appear. It is characterized by severe impairment in both intellectual and adaptive functioning.

A next generation sequencing analysis identified in an Iranian consanguineous family affected by ID a nonsense mutation in *CLIP1*, a gene that encodes a member of MTs plus-end tracking proteins, with a fundamental role in MTs dynamics regulation and MTs-mediated transport along axons and dendrites [161].

Kevenaar and colleagues found a causal link between homozygous nonsense mutations in kinesin-binding protein (KBP)/KIAA1279 and the neurological disorder Goldberg-Shprintzen syndrome (GOSHS), which is a form of intellectual disability combined also with microcephaly and axonal neuropathy [162]. KBP is a specific kinesin inhibitor that modulates MTs-based motility and depolymerizing activity of several kinesins. Indeed, kinesin motor proteins control both intracellular cargos transport and MTs cytoskeleton organization and the disruption of kinesin regulation by KBP causes severe cellular alterations during neurodevelopment leading to GOSHS neurological disorder.

Others kinesin family (KIF) members have been found to be related to ID; in fact, by next generation sequencing, mutations in *KIF4A* and *KIF5C* have been identified in ID patients [163]. To further confirm the link between *KIF* genes mutations and ID, in vivo functional studies on knockout mice have been performed. It emerged that, in addition to alterations in MTs dynamics and intracellular transport along MTs, *KIF4A* and *KIF5C* mutations lead to an imbalance between excitatory and inhibitory synaptic excitability. By means of whole exome sequencing of ID patients, de novo missense mutations also in *KIF1A* gene were discovered [164]. In vitro studies by MTs gliding assay confirmed the serious effects of *KIF1A* gene loss of function on axonal transport.

Mutations in *CHAMP1* gene also cause a form of ID associated to severe speech impairments [165,166,167]. CHAMP1 is a zinc finger protein involved in kinetochore-MTs attachment and in the regulation of chromosome segregation; ID-related *CHAMP1* mutations cause altered localization of CHAMP1 protein to chromosomes and to the mitotic spindle, impairing fundamental developmental processes.

Microdeletions incorporating the *KATNAL1* gene locus result in intellectual disability in humans [168]. This gene codes for the MTs severing protein KATNAL1. Mice carrying a loss of function allele in *KATNAL1* present specific behavioral deficits in circadian rhythms, sleep, anxiety, learning and memory. Furthermore, they present numerous brain structural abnormalities and defects in motile cilia [169].

Mutation of *MID2*, a gene coding for an ubiquitin ligase that regulates MTs dynamics, leads to misregulation of MTs organization and to the downstream disease pathology associated with X-linked intellectual disabilities [170]. Mid2 depletion led to stabilization of Astrin, a MTs organizing protein, during cytokinesis causing cytokinetic defects, multinucleated cells and cell death.

## 3. MTs Stabilizing Agents in NDDs

MTs interacting drugs have been proposed for the therapy of several NDDs, ranging from autism and schizophrenia, mainly due to their stabilizing properties, aiming to restore neuronal axonal transport and reduce tau phosphorylation. It should be noted that the concept of “MTs stabilization” has a proper meaning only when referring to in vitro condition. Concerning in vivo condition, the term “MTs stabilizing agents” has to be intended as something that acts on MTs dynamics slowing down the MTs turnover and depolymerization. Indeed, MTs maintain their dynamic regulation of growth/shrinkage but a delay in the time needed for their turnover renders them more stable, so sustaining intracellular cargo trafficking along MTs and maintaining synaptic contacts.

### 3.1. Brain-Penetrant Microtubule-Stabilizing Compounds: Epothilone D

Epothilone D (EpoD) is a taxol-related compound that interacts directly with tubulin to stabilize MTs [171,172]. EpoD can accumulate in brain tissues after crossing blood–brain barrier. Moreover, Epo D is currently used in clinical trials for the treatment of various tumors, indeed it blocks MTs dynamics and cell division at high dosages as reported by Wang and colleagues [173]. At nanomolar concentrations, however, this compound improves MTs density and axonal transport, reduces axonal dystrophy and enhances cognitive performance, without notable effects on viability and development in mouse [174]. In the STOP null mice, EpoD was found to have a beneficial effect on both synaptic transmission and behavior, reducing frequency of shifts between different spontaneous activities, such as feeding, walking and grooming [175].

### 3.2. Non-Taxane MTs Stabilizers: NAP and D-SAL

Activity-dependent neuroprotective protein (ADNP) and activity-dependent neurotrophic factor (ADNF) generate non-taxane MTs stabilizers such as NAPVSIPQ (NAP, davunetide) and all D-amino acid SALLRSIPA (D-SAL). ADNP has been associated with neuronal cell differentiation, maturation and neuronal plasticity [176]. ADNP down regulation in cell models resulted in MTs reorganization leading to altered cell morphology and reduced neurite formation [176]. In human, *ADNP* is mutated in autistic children with cognitive deficits [59,156,177] and is deregulated in schizophrenia [112,113]. MTs end binding (EB) proteins interact with NAP which showed a neuroprotective activity [67]. The EB proteins family, which consists of three members (EB1–3), is the core component of the MTs plus end tracking proteins (+TIPs) machinery which coordinates a network of dynamic proteins on the growing MTs plus-ends [178]. Furthermore, NAP enhances Tau-MTs binding under stress conditions and stimulated axonal transport [68,179,180]. In a *DISC1* mutated mouse model of schizophrenia, NAP treatment was shown to ameliorate behavior improving cognition and reducing anxiety [181]. Indeed, NAP treatment induces an improvement in daily activities in patients with schizophrenia in a phase II clinical trial [182]. Moreover, NAP protects from autophagy in a mouse model of schizophrenia enhancing the interaction between ADNP and the MTs-associated protein 1 light chain 3 β (LC3B) and restoring Becn1 mRNA levels in the hippocampus [183].

In a model of epileptic seizures, injection of NAP into the dentate gyrus of kainic acid-treated rats partially protected against CA3 neuronal death [184].

D-SAL competes with NAP binding to tubulin and prenatal treatment with NAP+D-SAL prevents developmental delay, glial deficit and learning impairment in Ts65Dn mice that are a well-characterized mouse model for Down syndrome [185].

### 3.3. Risperidone

Risperidone is a second-generation antipsychotic drug, acting by blocking dopamine D2 and serotonin 5-HT2A receptors, with consequent reduction of dopaminergic neurotransmission in the mesolimbic pathway. As NAP, risperidone was found to interact with EB1-EB3 protein family and to compete with NAP binding on EB3. Risperidone improves the neurocognitive functions in patients with schizophrenia [186] and is used in autistic patients for the treatment of associated symptoms as disruptive behavior and hyperactivity [187]. Similar to NAP, risperidone ameliorated object recognition deficits in the mutated DISC1 mouse model, although without the anxiolytic effect. NAP and/or risperidone treatment generated a down-regulation of Forkhead-BOX P2 (Foxp2) in the hippocampus of the *DISC1* mutated mice. This gene regulates DISC1 and is associated with human ability to acquire a spoken language. Thus, language disturbances and cognitive impairments in schizophrenia may be contrasted with the combination of NAP and risperidone [181]. Long-term administration of Risperidone was effective in alleviating behavioural alterations that mimic schizophrenia negative symptoms and partially ameliorate some cognitive defects in STOP null mice [188]. These mice present a disruption of MTs-associated protein 6 (*MAP6*) gene and are used as NDDs model.

### 3.4. New Experimental MTs-Acting Compounds

Currently several compounds are under research trial to study their potential use as MTs modulating agents in the context of neurodevelopmental disorders.

One of these compounds is melatonin, a hormone involved in the regulation of circadian rhythms, blood pressure, seasonal reproduction [189]. Melatonin can also act as neuronal antioxidant agent able to prevent MTs oxidative damage. Indeed, oxidative stress, by modifying β-tubulin and MTs-MAP2 binding, causes MTs depolymerization, consequent loss of polarization and apoptotic cell death [190]. Melatonin is able to increase MAP2 levels thus increasing MTs polymerization and dendrite stabilization [191,192]. Several data support the potential use of melatonin as MTs stabilizing agents in the treatment of depression and anxiety associated to schizophrenia [102,193].

Another interesting compound is a synthetic neurosteroid called MAPREG, a MTs-associated protein/neurosteroidal pregnenolone. It has been studied in the context of schizophrenia-associated depression to prevent branching reduction and synaptic contact loss [194]. In rodents MAPREG was able to protect them from developing a depressed state. Furthermore, it prevented the loss of α-tubulin acetylation and sleep disturbances following psychosocial stress [195]. By binding MAP2, MAPREG stimulates MTs assembly and promotes neurite growth [196].

Also lithium can act on MTs and have a beneficial role in cytoskeleton regulation in mood disorders. Indeed, it has been shown to act as a Gsk3 inhibitor, thus decreasing phosphorylation of tau and MAP1B finally leading to MTs remodeling [197].

Several studies report encouraging data regarding the possible use of calpain inhibitors to prevent LIS1 degradation in lissencephaly [198,199]. Calpain is a protease promoting LIS1 protein degradation. Calpain inhibitors protect LIS1 from proteolysis thus increasing LIS1 protein levels. LIS1 regulates cytoplasmic dynein function and localization. *LIS1* gene mutations, leading to absence, low levels or low activity of LIS1 protein, cause impaired MTs trafficking and neuronal migration during neurodevelopment. Toba and colleagues demonstrated that perinatal treatment with SNJ1945, a calpain inhibitor able to cross the blood brain barrier, can rescue LIS1^+/−^ mice defects. It stimulated network formation, receptor distribution, recovered retrograde transport and improved behavioral performance [199]. Yamada and colleagues demonstrated that treatment of pregnant Lis1^+/−^ dams with calpain inhibitor ALLN rescued apoptotic neurons death and neuronal migration defects in Lis1^+/−^ offspring [198].

Finally, in recent years several blood brain barrier penetrant-molecules have been identified that exhibit MTs-stabilizing activity [200]. These compounds, including triazolopyrimidines, phenylpyrimidines and other related heterocyclic molecules, are non-natural small molecules and have simple structure and favorable pharmacokinetic properties, such as oral bioavailability [201,202,203]. The mechanism of action of these compounds has not yet been completely defined at the molecular level; Zhang and colleagues demonstrated that they are able to block the binding of the tubulin polymerization inhibitor vinblastine. They have a taxol-like effect but act also on taxol-resistant cells and have been extensively studied as anticancer drugs. Now these small molecules are studied as drug candidates also for neurodegenerative diseases, such as Alzheimer’s disease and tauophaties [203], but it is conceivable to imagine their future application also in the context of NDDs.

## 4. Conclusions

Centrosome, MTs and MTs-related proteins are essential players in all the steps of brain development, from proliferation and migration to differentiation and synaptic network formation. Increasing data demonstrate that MTs-dependent mitotic and post-mitotic processes are major contributors of cortical malformations at the basis of NDDs. Brain formation during neurodevelopment requires the correct coordination of several developmental steps, regulated by both a precise timing and positioning. The disruption of one of these specific steps is sufficient to impair to whole process. Depending on the neurodevelopmental step involved, different NDDs can arise. The study of molecular mechanisms at the basis of several NDDs that still today have an unknown etiology or linked to genes with unknown functions is fundamental in order to find new pharmacological target for future therapies. MTs targeting therapies could be able to improve or even prevent some structural and functional alterations linked to NDDs. Currently only few MTs targeting compounds have been tested on humans in clinical trials but increasing number of new compounds are being tested in experimental studies in both in vitro and in vivo models and promising results are emerging. The possibility to act on MTs for pharmacological intervention in NDDs is progressively becoming a solid opportunity.

## Figures and Tables

**Table 1:** MTs and MTs regulatory protein and gene alterations associated with Autism (ASD).

Genes/Protein	Effects	Ref.
*ADNP*	*ADNP* mut cause intellectual disability, facial dysmorphisms	[66,67,68,69]
	ADNP^−/−^ mice are not viable due to failure of neural tube closure	
	ADNP^−/+^ mice present tauopathy and neuronal cell death, deficit in social behavior and object recognition	
	ADNP knock down in cells provokes decreased number of neurites and decreased number and size of embryoid bodies	
Slit/Robo	Increased expression of *Slit 1*, decreased expression of *ABL1* and *Cdc42* are associated to alteration in neurite branching and axon pathfinding	[70,71]
JAKMIP1	JAKMIP1 overexpression causes the formation of tight and stable MTs boundles in human cell lines	[72,73]
	JAKMIP1^−/−^ mice present ASD-like behaviors	
STOP/MAP6	STOP/MAP6^−/−^ mice present synaptic abnormalities and ASD-like behavioral deficits (impairments in maternal care, social behavior and reduced cognitive performance)	[74,75,76,77,78]
	Reduced plasma levels of STOP/MAP6 protein in ASD patients	
KIRREL3/MAP1B	KIRREL3/MAP1B interaction involved in ASD pathogenesis	[79,80,81,82]
AUTS2/Rac1	*AUTS2* mut neurons display abnormal morphology during migration and reduced activity of JNK	[83,84,85,86]
YWHAE	*YWHAE* duplication associated to ASD	[87,88]
	YWHAE regulator of neurite formation acting on Dcx	
MARKs	Dysregulation of MARKs has been linked to ASD	[89,90,91]
	MARKs regulate MTs dynamics during cell polarity, migration and vesicular transport	
KATNAL2	*KATNAL2* de novo mut associated to ASD in human	[92,93]
	KATNAL2^−/−^ mice present reduced neurite branching and length	

**Table 2:** MTs and MTs regulatory protein and gene alterations associated with schizophrenia.

Genes/Protein	Effects	Ref.
α-tubulin and β-tubulin	Altered cytoskeletal organization	[100,101,102]
*DISC-1*	Mutation S704C confers susceptibility to schizophrenia in humans	[110,111]
*ULK4*	Ulk4^−/−^ mice showed low levels of acetylated α-tubulin	[103]
	*ULK4* gene deletions have been found in schizophrenic patients	
TTLL 11	Balanced chromosomal translocation combined with chromosomal micro-duplication is associated with increased schizophrenia susceptibility	[104,105]
MAP1B	Low immunoreactivity in hippocampal subiculum associated with altered cyto-architecture and neurotransmission deficits in individuals with schizophrenia	[81,82]
MAP2	Low immunoreactivity in brains of individuals with schizophrenia	[107,108]
MAP6	Neuronal transport defects	[78,109]
	Deletion of gene causes altered mood and cognitive performance in mice models of schizophrenia	
ADNP	ADNP protein deregulated in postmortem hippocampi of schizophrenia patients	[112,113]
	ADNP involved in autophagy regulation	

**Table 3:** MTs and MTs regulatory protein and gene alterations associated with lissencephaly.

Genes/Protein	Effects	Ref.
LIS1	Loss of lissencephaly-1 (LIS1) protein is a major cause of lissencephaly	[143,144,145]
	Mutations on *LIS1* gene cause alterations in neuronal migration, in MTs network organization and intracellular transport	
*DCX*	*DCX* gene mutations cause neuronal migration abnormalities by altering MTs dynamics	[146,147,148,149,150]
	*DCX* gene has been associated with lissencephaly and subcortical band heterotopia	
*TUBA1A*	Mutations in the *TUBA1A* gene have been associates with type I lissencephaly	[151,152]
*TUBB2B*	Mutations in *TUBB2B* gene cause asymmetrical bilateral polygyria	[153]
*RELN*	*RELN* gene mutations cause loss of reelin protein, leading to alterations in neuronal migration and positioning in the developing brain	[53]
